# Re: Teenage Pregnancies in Nepal - The Problem Status and Socio-Legal Concerns – Letter to The Editor

**DOI:** 10.31729/jnma.4570

**Published:** 2019-08-31

**Authors:** Samata Nepal, Alok Atreya, Tanuj Kanchan

**Affiliations:** 1 Department of Community Medicine, Lumbini Medical College, Palpa, Nepal; 2 Department of Forensic Medicine and Toxicology, Lumbini Medical College, Palpa, Nepal; 3 Department of Forensic Medicine & Toxicology, All India Institute of Medical Sciences, Jodhpur, India

Dear Editor,

We would like to thank the editor for giving us an opportunity to respond to the letter on our article entitled “Teenage Pregnancies in Nepal - The Problem Status and Socio-Legal Concerns”.^1^ We would also like to thank the readers for their interest in our paper and taking time to express their concerns.

Nepal is one of the popular tourist destinations. The study site for the present study was Manipal Teaching Hospital, Pokhara.  All patients who provide their permanent address in Nepal are presumed as locals, while for the patients who state their address other than in Nepal, they are considered non-Nepalese nationals. The Manipal Teaching Hospital is mostly visited by locals for management of various ailments. The hospital usually attends to foreigners who get ill during trekking in the mountains, or injure themselves during the adventurous sports including paragliding, zip-flying and honey hunting to mention a few.

The aforementioned study was conducted with a primary objective to find out the incidence of teenage pregnancies in Pokhara region of Nepal, and to discuss the associated medical, legal and social aspects. The address of the study participants was noted from the admission forms and the patient and caregivers were interviewed in local Nepali language. All the participants were permanent residents of Nepal with a local address within Nepal. Besides, the authors observed fluency in the conversation while interviewing the patients/ patient's caregivers in Nepali. Although, nationals from Nepal and India share similar cultural and socio-economic background, the Indian nationals lack the fluency of the Nepali language to a certain extent. Thus, there was nothing suggestive of the caregiver being non-Nepalese.

Pokhara is almost 180 kilometers from the nearest Indian border. Due to difficult geography and road conditions, it is unlikely that Indian teenage mothers would come this far only to deliver her child. Even if we consider the remote possibility of an Indian teenage already residing in Nepal, being included as one of the participant in the study, it still remains a teenage pregnancy with delivery occurring in Nepal. Nationality was not a variable for the present study, which was aimed at raising the attention of all stakeholders towards the problem of teenage pregnancies and associated issues.
